# Seroprevalence of *Echinococcus* spp. and *Toxocara* spp. in Invasive Non-native American Mink

**DOI:** 10.1007/s10393-020-01470-3

**Published:** 2020-01-27

**Authors:** Marta Kołodziej-Sobocińska, Emília Dvorožňáková, Zuzana Hurníková, Katarína Reiterová, Andrzej Zalewski

**Affiliations:** 1 Mammal Research Institute, Polish Academy of Sciences, Stoczek 1, 17-230 Białowieża, Poland; 2 Institute of Parasitology, Slovak Academy of Sciences, Hlinkova 3, 040 01 Kosice, Slovak Republic

**Keywords:** Invasive species, Seroprevalence, Zoonoses, Paratenic host, Echinococcosis, Toxocarosis

## Abstract

Invasive non-native species can become reservoirs of zoonotic pathogens and cause their spread during colonization, increasing the risk of zoonoses transmission to both wild hosts and humans. American mink (*Neovison vison*) are considered an important invasive mammal species responsible for carrying endoparasites. The aim of our study was to evaluate the role of feral American mink as a possible transmission vector of *Echinococcus* spp. and *Toxocara* spp. in wildlife. We analysed the frequency of American mink exposure to both parasites, the spatial distribution in Poland, and the variability over time on the basis of specific antibody presence using ELISA and Western blot. Alimentary tract analyses revealed that American mink do not serve as definitive hosts for these parasites. Altogether, 1100 American mink were examined. The average seropositivity for American mink was 14.2% for echinococcosis and 21.7% for toxocarosis; dual-seropositivity was detected in only 6.0%. Seroprevalence of both parasites differed between study sites and significantly increased over time in *Toxocara* spp. Thus, our study revealed that free-living American mink are exposed to parasites and likely to be involved in the maintenance of both *Echinococcus* spp. and *Toxocara* spp. in the wild as paratenic hosts.

## Introduction

Invasive non-native species are among the greatest threats to biodiversity, often causing a decrease in native species number and density in ecosystems (Vitousek et al. 1996). Among many others, one of the negative impacts of invasive species on ecosystems is their role as additional transmission vectors or reservoirs of native parasites and pathogens. Raccoon dogs (*Nyctereutes procyonoides*), American mink (*Neovison vison*), and raccoons (*Procyon lotor*), for instance, may be involved in the maintenance and transmission of many diseases across Europe, including those with zoonotic potential such as *Trichinella* spp., *Alaria* spp., *Echinococcus multilocularis*, and *Toxocara* spp. (Duscher et al. 2017; Hurníková et al. 2016; Laurimaa et al. 2016). The transmission rate of pathogens may be enhanced when introduced non-native invasive species reach high densities in newly colonized ecosystems (Kelly et al. 2009; Strauss et al. 2012; Carolus et al. 2019). Likewise, this increases the risk of disease occurrence in wild native hosts, domestic animals, and potentially humans (Carolus et al. 2019).

Pathogens like *Echinococcus* spp. and *Toxocara* spp. are important zoonotic agents with worldwide distributions (Jorgensen et al. 2008; Kern et al. 2003; Nahorski et al. 2013; Overgaauw and Nederland 1997). Alveolar echinococcosis (AE) occurs in at least 42 countries in the northern hemisphere (Kern et al. 2003; Nahorski et al. 2013). The larval form of *E. multilocularis* is the causative agent of AE in humans. Transmission of AE—a life-threatening helminthic zoonosis—to humans occurs when tapeworm eggs are accidentally ingested. Then, larvae settle in the liver and proliferate, and can also metastasize to more distant organs like the brain. The fatality rate for untreated human AE exceeded 90% within 10 years (Kern et al. 2003). European cases of AE have been registered since 1982 (Kern et al. 2003). In Poland, in particular, a total of 121 cases of AE have been confirmed (Nahorski et al. 2013), which puts Poland in the fourth place among all European counties. In neighbouring countries, like Germany, Slovakia, and the Czech Republic, AE is also a serious epidemiological problem (Jorgensen et al. 2008; Antolová et al. 2014; Kolarova et al. 2015). Knowledge about the reservoir of AE in wildlife focuses mainly on red fox (*Vulpes vulpes*) infection (Bagrade et al. 2016; Karamon et al. 2014; Miller et al. 2016; Umhang et al. 2016). Thus, it is thought that human AE is mainly present in areas with a large prevalence of infected red foxes (Nahorski et al. 2013; Schweiger et al. 2007). Nematode parasites of the genus *Toxocara* also cause severe diseases in humans (Chen et al. 2018), as people can acquire infection by, among other methods, ingestion of embryonated eggs present in contaminated soil or food. *Toxocara* spp. larvae migrate to various body organs, such as the liver, heart, lungs, kidneys, brain, muscles, or eyes, which causes a broad range of clinical symptoms.

Both echinococcosis and toxocarosis can be diagnosed with ELISA and Western blot. The use of serological data to study infection in wildlife is widely accepted (Fillaux and Magnaval 2013; Lassen et al. 2016; Pagnozzi et al. 2016). Seroprevalence studies of infected wild animals can be a good, easy, and cheap way to determine the occurrence of these zoonotic parasites in the environment. Serological methods offer a sensitive and relatively specific alternative to necropsies or digestion methods. The usefulness of both ELISA and Western blot was confirmed, for example, in *T. canis* and *T. leonina* diagnoses in experimentally infected paratenic hosts (Klockiewicz et al. 2019a). No false-positive/false-negative results were stated in this study. In cases when blood samples are not available, muscle juice acts as the equivalent to serum for the detection of specific antibodies for *E. multilocularis* (Gottsteina et al. 2014) or *Trichinella spiralis* (Beck et al. 2005; Møller et al. 2005; Nöckler et al. 2005). An advantage of using muscle juice includes the long-term storage of samples collected for epidemiological studies, and it can also be a good alternative to testing samples from wild animals.

Knowledge about the presence of *Echinococcus* spp. and *Toxocara* spp. in wildlife is still sparse, and there is a need to supplement it with new data about possible reservoir hosts, sites of occurrence, and the importance of introduced species in the maintenance of these parasites in the environment. The prevalence of echinococcosis has mainly been studied in the red fox, and in Poland the highest proportion of infected individuals has been recorded in eastern regions (Karamon et al. 2014). However, other predators, including invasive non-native species, may participate in maintaining high abundances of the parasite in wildlife, e.g. the raccoon dog (Bagrade et al. 2016; Machnicka-Rowińska et al. 2002). Carnivorous mammals are hosts for both *Echinococcus* spp. and *Toxocara* spp. parasites (Bagrade et al. 2016; Holland 2017). Definitive hosts, such as red foxes, raccoon dogs, and wolves, shed parasite eggs into the environment and are important vectors in disease transmission, not only to other wild hosts, but also to humans who may accidentally ingest invasive forms of the parasites. Intermediate and/or paratenic hosts allow both parasites to complete their life cycles because larval forms settled in these hosts’ tissues are the source of infection for definitive hosts—the carnivores preying upon them (Eckert and Deplazes 2004). Thus, intermediate and paratenic hosts, such as rodents and smaller predators, may enhance the probability of disease transmission (Wobeser 2006). Therefore, understanding parasitic zoonoses also requires data on the invasive mammal species’ infection status, because these species may serve as hosts for parasites with zoonotic potential.

The American mink has been recognized as one of the most invasive alien mammal species and has significant impacts on ecosystems, reducing the number of native birds and mammals (Nentwig et al. 2010; Niemczynowicz et al. 2017; Brzeziński et al. 2020). The species originates from North America and has been introduced to many countries on three continents, mainly via breeding farms for fur production (Bonesi and Palazon 2007). In Europe, the American mink was introduced in the 1930s and has colonized 28 countries (Bonesi and Palazon 2007). In Poland, the feral population established itself in the early 1980s and over the next 40 years colonized nearly the entire country except the mountains and uplands of south-eastern Poland (Zalewski et al. 2010; Brzeziński et al. 2019). In colonized areas, American mink population levels reach densities as high as 10–12 individuals per 10 km of river (Bartoszewicz and Zalewski 2003; Brzeziński et al. 2019). The species is a medium-sized mustelid and is a highly adaptive, opportunistic, semi-aquatic predator, inhabiting a variety of riparian habitats (Kauhala 1996; Sidorovich 2000; Brzeziński et al. 2018b; Zalewski et al. 2009). This wide range of occupied habitat is related to the species’ generalist diet composition; American mink hunt varied prey, including both aquatic (mainly crayfish, fish, and frogs) and terrestrial (birds and rodents) species (Jędrzejewska et al. 2001; Zalewski and Bartoszewicz 2012). Dietary composition varies greatly between seasons and localities and has also changed over the period of American mink invasion (Jędrzejewska et al. 2001; Bartoszewicz and Zalewski 2003; Sidorovich et al. 2010; Brzeziński et al. 2018a). The carnivore typically lives no more than 6 years, but the mortality rate is high, especially in subadult mink (Bonesi et al. 2006). American mink may be killed and eaten by many larger predators, including dogs (*Canis lupus familiaris*) and wild carnivores like the Eurasian lynx (*Lynx lynx*), red fox, and wolf (*Canis lupus*) (Bryan et al. 2006; Errington 1937; Odden et al. 2006; Sepúlveda et al. 2014).

Data on the number of endoparasite species and infection rates in introduced ranges of American mink have up until recently been limited (Shimalov and Shimalov 2001a; Torres et al. 2003, 2008). Recent data from Poland have shown that newly established populations have much lower endoparasite abundances than older populations, but that these abundances increased within a relatively short period of approximately 20 years (Kołodziej-Sobocińska et al. 2018). On this basis, it can be assumed that in new territories where populations were established, the American mink potentially enhances risk of zoonotic transmission to humans. This was recently proven by our detection of three *Trichinella* species (*T. spiralis*, *T. britovi*, and *T. pseudospiralis*) in the American mink in its introduced range in Poland (Hurníková et al. 2016). Neither *Echinococcus* spp. nor *Toxocara* spp. infection (adult parasites and/or eggs) have been confirmed in American mink (Kołodziej-Sobocińska et al. 2018; Martínez-Rondán et al. 2017; Shimalov and Shimalov 2001a; Torres et al. 2003, 2008). In addition, the sole published experimental study found that after oral inoculation with *Echinococcus multilocularis* protoscoleces, no adult tapeworms were recovered from American mink intestines, and the authors concluded that American mink cannot serve as a definitive host for *E. multilocularis* (Ooi et al. 1992). Moreover, a recent experimental study done on farm American mink confirmed that this species may serve as a paratenic host for *Toxocara canis* and *Toxascaris leonina* (Klockiewicz et al. 2019a). Thus, American mink should be further investigated to receive more data about their involvement in maintaining zoonotic parasites in the environment, though it can be assumed that this species serves as a paratenic host for both parasites.

The aim of our study was to evaluate the frequency of American mink exposure to two zoonotic parasites—*Echinococcus* spp. and *Toxocara* spp.—and determine the role of this invasive species in the maintenance of both parasites in the environment. In this study, we analysed seroprevalence by detecting specific anti-*Echinococcus* spp. and anti-*Toxocara* spp. antibodies (using ELISA confirmed by Western blot) at various sites in Poland. We analysed the frequency of American mink exposure to both parasites, the spatial distribution of seropositive individuals in Poland, and the variability in seropositivity of both parasites over time. We first hypothesized that seropositivity will increase over time, a feat that is connected to patterns of parasite acquisition by non-native invasive mammal species during the colonization of new territories. Secondly, we predicted that the spatial pattern of prevalence in American mink will be related to the spatial pattern in red foxes, but also differ among various locations in Poland. Finally, the third part of our hypothesis claimed that this non-native invasive mammal species serves as a paratenic host for *Echinococcus* spp. and *Toxocara* spp. and may play a role in the maintenance and spread of zoonotic parasites on the colonized area.

## Methods

### Study Area and Sample Collection

**Figure 1:**
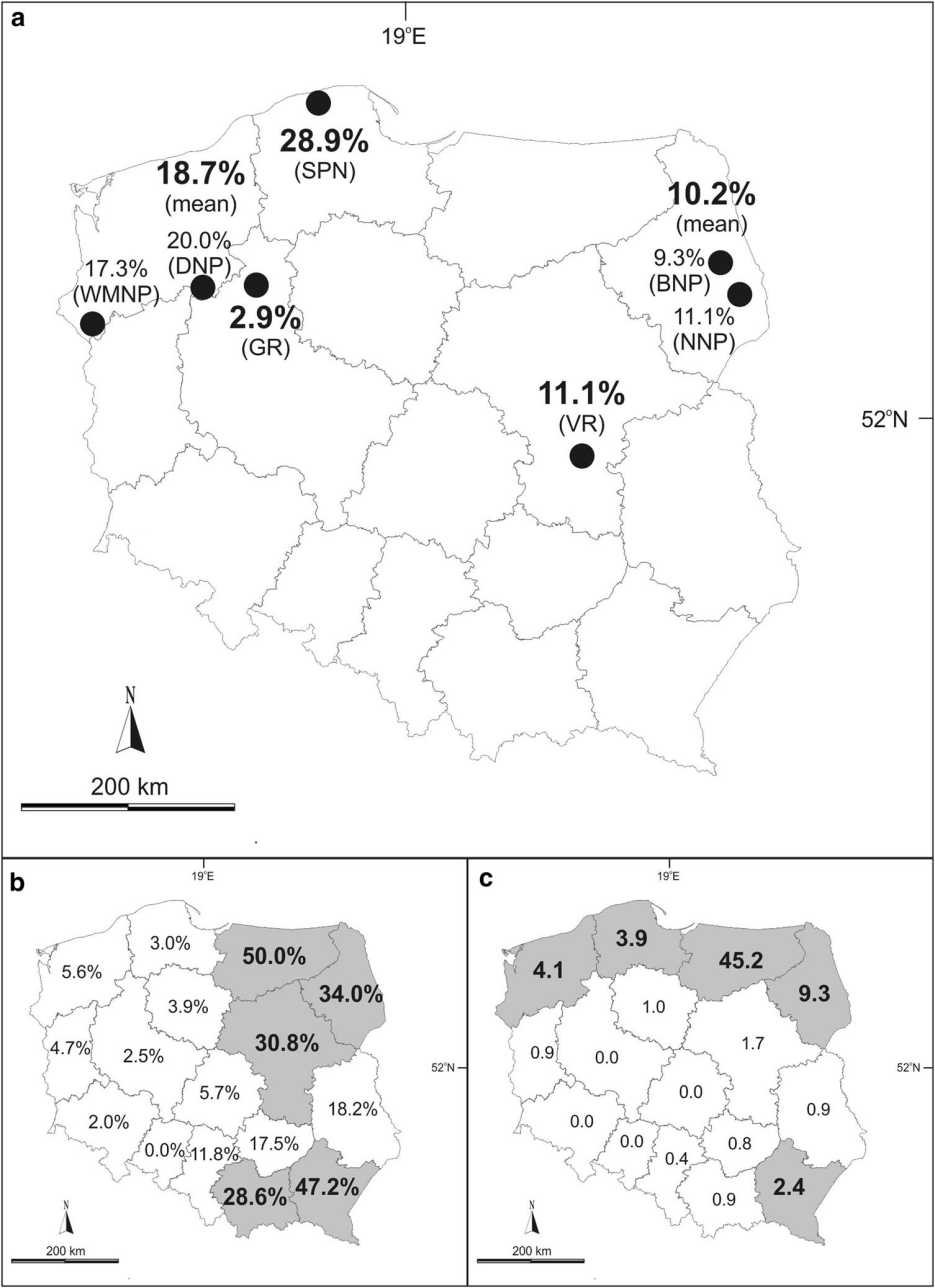
Comparison of the occurrence of echinococcosis in various hosts in Poland: **a** seroprevalence in the American mink (this study); **b** prevalence in the red fox (Karamon et al. 2014); **c** number of human cases per million inhabitants (data on inhabitant numbers in provinces were taken from the Central Statistical Office of Poland) (Nahorski et al. 2013). Study sites are marked with black dots: WMNP—Warta Mouth National Park, GR—Gwda River, DNP—Drawa National Park, SNP—Słowiński National Park, VR—Vistula River, BNP—Biebrza National Park, NNP—Narew National Park. Seroprevalence of American mink for each studied province is given; in the case of data being made available from two study sites located in one province, the mean value is provided (**a**). Provinces marked with grey colour indicate places where the highest prevalence of echinococcosis was stated; for red fox above 25% (**b**) and for humans above 2 human cases per million inhabitants (**c**).

We collected 1100 American mink individuals between 2006 and 2017. They were trapped at seven study sites: three in eastern Poland (Biebrza National Park—BNP, Narew National Park—NNP, and Vistula River—VR) and four in western Poland (Warta Mouth National Park—WMNP, Drawa National Park—DNP, Gwda River—GR, and Słowiński National Park—SNP) (see Fig. 1a). In all sites, carcasses were collected during American mink eradication carried out by the staff of national parks or bird conservation organizations as part of several bird conservation projects. Thus, based on Resolution No. 22/2006 of the National Ethics Committee for Animal Experiments, no separate approval from the Local Ethics Committee for Animal Experimentation was needed to obtain tissues samples from carcasses and conduct the study. Muscle samples (1–5 g) from the diaphragm and hind limbs as well as alimentary tracts were collected during autopsies of American mink and frozen at − 20°C until examination.

### Parasitological Examination

Parasitological examination was performed using a standard scraping technique with slight modifications (e.g. Eckert et al. 1984) on 549 randomly selected American mink individuals (50% of all studied individuals). The alimentary tracts were isolated and examined for the presence of internal parasites, which included, among others, *E. multilocularis* and *Toxocara* spp. In brief, each alimentary tract was divided into four parts: stomach, duodenum, small intestine, and large intestine. Each part was then cut longitudinally, and mucosal scrapings were collected. Material from each part of the alimentary tract was examined under a binocular or microscope. Worm burden was determined separately for each tract part.

### Preparation of Used Antigens

#### Somatic Antigen *Echinococcus multilocularis*

Metacestodes of *E. multilocularis* were maintained in Mongolian gerbils (*Meriones unguiculatus*) by intraperitoneal serial passage of protoscoleces. After careful dissection from the peritoneal cavity, the metacestodes were washed several times in PBS (pH 7.2) containing antibiotics and antimycotics and were then pressed through metal mesh screens. After several washing steps, a suspension of protoscoleces free of flocculent tissue debris was obtained. Then, protoscoleces were homogenized in an ice-cold glass (SONOPULS ultrasonic homogenizer HD3100, Bandelin, Germany). The homogenate was centrifuged at 4500 *g* for 5 min at 4°C, and the supernatant was concentrated in 3000 MWCO Vivaspin tubes (Sartorius, Goettingen, Germany) at 4000 *g* for 100 min at 4°C. The protein concentration of the larval antigen was measured using the Bradford protein assay (Bio-Rad Laboratories, Munchen, Germany).

#### Larval Excretory–Secretory Antigen *Toxocara canis*

The excretory–secretory antigen of *T. canis* was prepared according to the method of de Savigny (1975). Larvae were cultivated in RPMI 1640 (Sigma-Aldrich, Hamburg, Germany) medium modified with 20 mM HEPES and l-glutamine, supplemented with penicillin/streptomycin (100 IU, 100 μg/ml) (Sigma-Aldrich, Hamburg, Germany). Larvae were maintained in sterile 25-ml tissue culture flasks (Falcon, Durham, USA) at a concentration of 10^3^ larvae/ml and incubated continually for a long term at 37°C under a 5% C0_2_ and 95% atmosphere humidity. The culture medium was replaced at 5-day intervals after assessing the viability of larvae. Starting at the third week, the collected pooled medium (from 3 weeks) was concentrated in 3000 MWCO VIVASPIN tubes (Sartorius, Goettingen, Germany) at 4000 *g* for 100 min at 4°C. The protein concentration of the larval antigen was measured using the Bradford protein assay (Bio-Rad Laboratories, München, Germany).

### Detection of Specific Anti-*Echinococcus* spp. and Anti-*Toxocara* spp. Antibodies from Muscle Samples

Muscles were melted and muscle juice was collected from the samples. The specific detection of anti-*Echinococcus* spp. and anti-*Toxocara* spp. antibodies in the muscle juice of American mink—using a somatic antigen from *Echinococcus multilocularis* protoscoleces and excretory–secretory (E/S) antigen of *Toxocara canis* larvae—was conducted with an indirect enzyme-linked immunosorbent assay (iELISA) according to Havasiová-Reiterová et al. (1995) with slight modifications. *Toxocara canis* excretory–secretory antigen (E/S antigen) is highly specific, can detect both *T. canis* and *T. cati* antibodies, and shows no cross-reactivity with other ascarid species (Cuellar et al. 1995; Krucken et al. 2017). Similarly, *Echinococcus multilocularis* antigen can detect both *E. multilocularis* and *E. granulosis* antibodies. The *E. multilocularis* cestode is principally maintained in sylvatic cycles, and *E. granulosus* is perpetuated by domestic animals (Thompson 2013). The iELISA was validated with samples from dogs positive with echinococcosis as positive controls (*n* = 5, *E. multilocularis* was confirmed by nested polymerase chain reaction (PCR); Antolová et al. 2009) and toxocarosis (*n* = 5, puppies positive for *T. canis* helminths) and parasite-free dogs (*n* = 3) as negative controls. Anti-dog IgG (Sigma-Aldrich, Hamburg, Germany) was used for the detection of antibodies in American mink. The efficiency of anti-dog IgG conjugate for the detection of mink-specific IgGs was validated by comparison with the multispecies conjugate—Protein G Peroxidase from *Streptococcus* sp. (Sigma-Aldrich, Hamburg, Germany). The test was set up after being calibrated with E/S antigen (diluted at 1, 2, 4, 10 µg/ml), muscle juices (undiluted, 1:10, 1:100), and anti-dog IgG conjugate (1:20,000 and 1:30,000). The best signal was chosen to detect anti-parasite antibodies in American mink. Cut-off values (optical density (OD) = 0.500 for anti-*Echinococcus* spp. antibodies, OD = 0.500 for anti-*Toxocara* spp. antibodies) were estimated as the mean absorbance of the negative controls plus three times the standard deviation (SD). We used a generalized linear model (GLM) with Gaussian family to compare OD value of anti-*Echinococcus* spp. antibodies for four classes of co-infection: individuals negative for *Echinococcus* spp. (Neg1), individuals negative for *Echinococcus* spp. but positive for *Toxocara* spp. (Neg2), individuals positive for *Echinococcus* spp. but negative for *Toxocara* spp. (Pos1), and individuals positive for both parasites (mixed infection) (Pos2). A similar GLM was used to analyse OD values of anti-*Toxocara* spp. antibodies.

Briefly, *E. multilocularis* and *T. canis* antigens, diluted to 2 µg/ml carbonate buffer (pH 9.6), were bound to the microtiter plates (Nunc, Thermo Fisher Scientific, Roskilde, Denmark) overnight at 4°C. After triple-washing of wells with phosphate-buffered saline (PBS, pH 7.2) with 0.5% Tween 20 (PBS-T), non-specific bonds were blocked with 0.5% skimmed milk PBS after 1 h of incubation at room temperature. After triple-washing with PBS-T, the muscle juice samples diluted at 1:100 were incubated for 1 h at 37°C. All samples were examined in duplicate. After the washings, bound antibodies were detected through incubation for 1 h at 37°C with horseradish peroxidase-conjugated rabbit anti-dog IgG (Sigma-Aldrich, Hamburg, Germany) diluted at 1:20,000. After final washings, the substrate *o*-phenylenediamine (Sigma-Aldrich, Hamburg, Germany) at 0.05 mol/l in citrate buffer (pH 4.7) with 0.005% H_2_O_2_ was used to trigger a colour. The reaction was stopped by 1 M H_2_SO_4_ after a 15-min incubation at room temperature in the dark. The optical density was measured at 492 nm (Multiskan Reader, Thermo Fisher Scientific, Vantaa, Finland).

To analyse the spatiotemporal variation in seroprevalence, we used a generalized linear model (GLM) with binomial error distribution. The effect of American mink sex, collection site, and year of American mink collection on the presence of both parasites were analysed in the model. All models were computed in the ‘lme4’ package in R (R Core Team 2016).

### Western Blot

Samples of 56 American mink, 28 positive for *Echinococcus* antibodies and 28 positive for *Toxocara* antibodies by ELISA, were examined by the Western blot technique. Electrophoresis (ELFO-SDS-PAGE) was performed using a Bio-Rad Mini Protein Slab Cell (Bio-Rad Laboratories, CA, USA) on a 12% SDS-polyacrylamide gel and 4% stacking gel under reducing conditions (Laemmli 1970). Protoscoleces antigen *Echinococcus multilocularis* and larval excretory–secretory *Toxocara canis* antigen were electrophoresed at 150 V and 90 mA for 60–70 min at room temperature. Low molecular weight markers (prestained SDS-PAGE standards, Bio-Rad) were included in each electrophoretic run. Following electrophoresis, proteins were transferred to a nitrocellulose (NC) membrane in Tris–glycine buffer (pH 8.8) at a constant voltage of 250 mA and 100 V for 90 min using a Bio-Rad Trans-Blot Cell. After blotting, the NC membrane was cut into 2.5-mm-wide strips and blocked with 5% non-fat milk powder in PBS (pH 7.2) for 60 min at 37°C. Meat juices diluted 1:50 with 3% non-fat milk in PBS were incubated over night at 37°C. The strips were washed three times with PBS-Tween 20 and reacted with horseradish peroxidase-conjugated anti-dog IgG (Sigma-Aldrich, Hamburg, Germany) in dilution of 1:500 for 1 h at 37°C with continuous shaking. Subsequently, the strips were washed three times with PBS-Tween 20, and bands were developed using 0.05% 4-chloro-1-naphthol in PBS (pH 7.2) and 0.03% hydrogen peroxide.

Positive control serum was obtained from a dog with confirmed *E. multilocularis* cysts localized in the liver. Likewise, we obtained *Toxocara*-positive control serum from a dog that had defecated adult toxocaral larvae after dehelmintization. Negative control serum was obtained from a parasite-free dog. Sera that showed reactions at 8; 16–18; 26–28 kDa bands were considered as *Echinococcus*-positive. Sera that showed reactions at 35, 38, 65–78 kDa bands were considered as *Toxocara*-positive.

## Results

**Figure 2:**
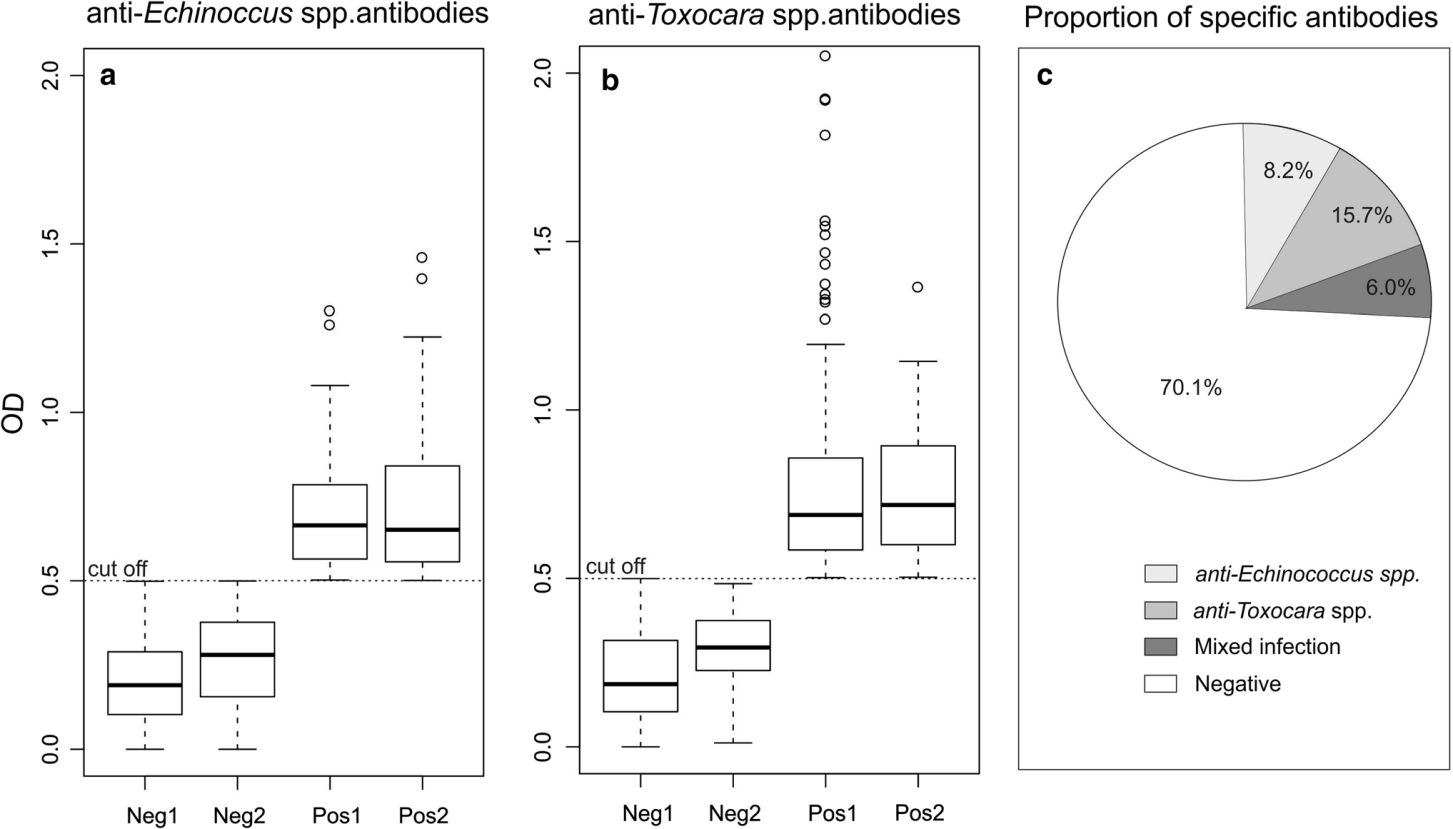
*Echinococcus* spp. and *Toxocara* spp. seropositivity of American mink. Optical density (OD) value of ELISA performed for: **a** individuals negative for *Echinococcus* spp.—Neg1, individuals negative for *Echinococcus* spp. but positive for *Toxocara* spp.—Neg2, individuals positive for *Echinococcus* spp. but negative for *Toxocara* spp.—Pos1, individuals positive for both parasites (mixed infection)—Pos2; **b** individuals negative for *Toxocara* spp.—Neg1, individuals negative for *Toxocara* spp. but positive for *Echinococcus* spp.—Neg2, individuals positive for *Toxocara* spp. but negative for *Echinococcus* spp.—Pos1, individuals positive for both parasites (mixed infection)—Pos2; **c** proportion of American mink seropositive for *Echinococcus* spp., *Toxocara* spp., and both parasites (dual infection) in Poland (*n* = 1100).

In total, 1100 muscle samples of American mink from seven feral populations were examined for the presence of specific anti-*Echinococcus* spp. and anti-*Toxocara* spp. antibodies. Additionally, 50% of the studied American mink (*n* = 549) were dissected and checked for intestinal parasite presence. Of 156 American mink seropositive for echinococcosis and 239 seropositive for toxocarosis, 89 (57%) and 90 (38%), respectively, were checked for intestinal parasite presence. Neither *Echinococcus* spp. nor *Toxocara* spp. adults were found. Total seroprevalence of *Echinococcus* spp. and *Toxocara* spp. in American mink was 14.2% and 21.7%, respectively (Table 1). On average, 6% of American mink from the seven sites were seropositive for both parasites, which suggests a mixed infection (Fig. 2c). There were no significant differences between OD values for seropositive animals regardless of whether mono- or mixed infections had been demonstrated (Fig. 2a, b; GLM for anti-*Echinococcus* spp. antibodies: *t* = 1.71, *p* = 0.087 and for anti-*Toxocara* spp. antibodies: *t* = − 0.21, *p* = 0.833).

**Table 1:** Seroprevalence of *Echinococcus* spp. and *Toxocara* spp. infection in feral American mink from seven study sites in Poland (*n* = 1100).

Study site	Number of studied animals (*n*)			*Echinococcus* spp. seroprevalence (%) (*n* positive in brackets)			*Toxocara* spp. seroprevalence (%) (*n* positive in brackets)		
	Females	Males	Total	Females	Males	Total	Females	Males	Total
WMNP	126	232	358	19.8 (25)	15.9 (37)	17.3	16.7 (21)	15.9 (37)	16.2
DNP	30	50	80	23.3 (7)	18.0 (9)	20.0	13.3 (4)	16.0 (8)	15.0
GR	18	17	35	0.0 (0)	5.9 (1)	2.9	61.1 (11)	70.6 (12)	65.7
SNP	20	32	52	40.0 (8)	21.9 (7)	28.9	55.0 (11)	34.4 (11)	42.3
VR	5	13	18	0.0 (0)	15.4 (2)	11.1	40.0 (2)	84.6 (11)	72.2
BNP	30	78	108	3.3 (1)	11.5 (9)	9.3	10.0 (3)	20.5 (16)	17.6
NNP	210	239	449	10.0 (21)	12.1 (29)	11.1	20.5 (43)	20.5 (49)	20.5
Total	439	661	1100	14.1 (62)	14.2 (94)	14.2 (156)	21.6 (95)	21.8 (144)	21.7 (239)

WMNP, Warta Mouth National Park; GR, Gwda River; DNP, Drawa National Park; SNP, Słowiński National Park; VR, Vistula River; BNP, Biebrza National Park; NNP, Narew National Park; n, sample size.

**Figure 3:**
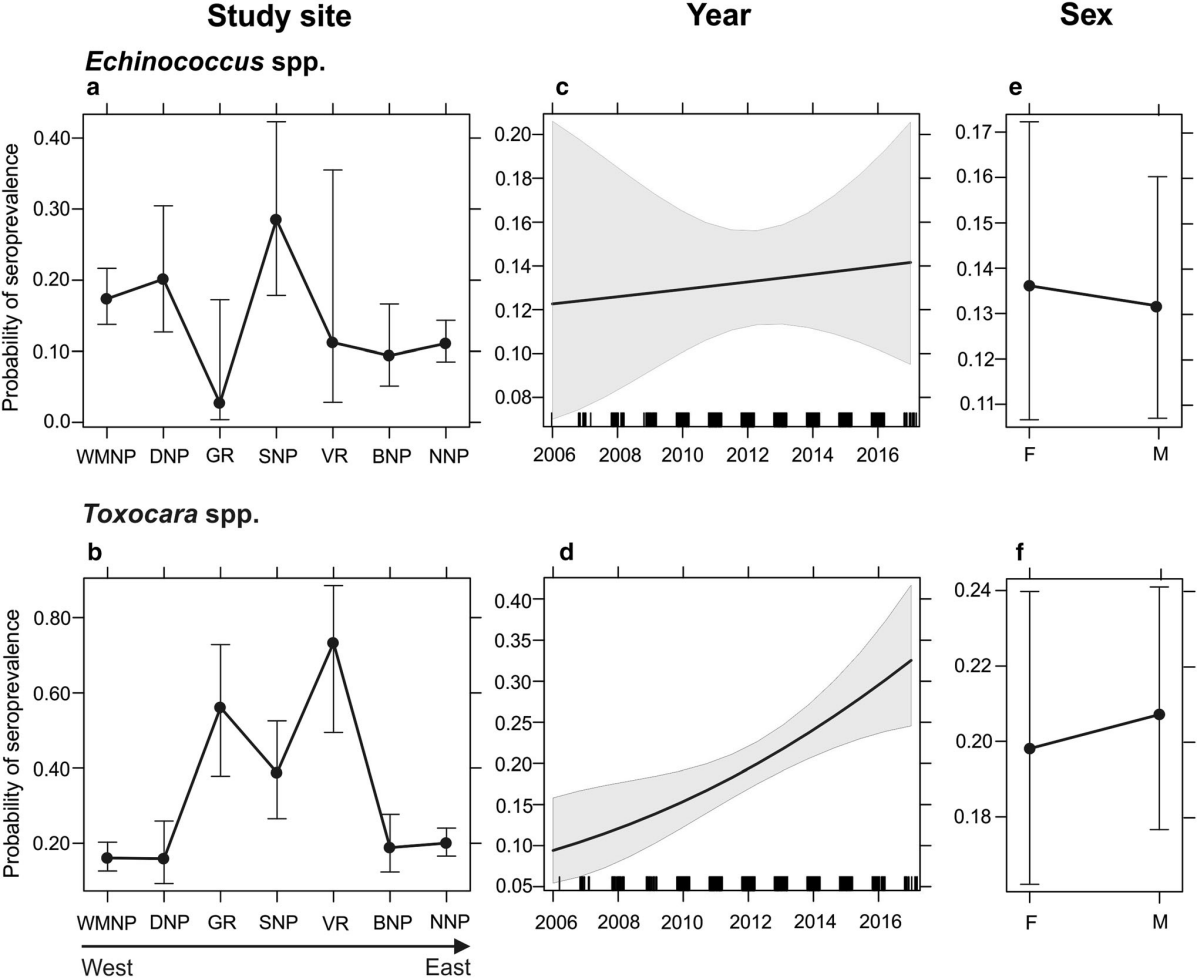
The seroprevalence of *Echinococcus* spp. and *Toxocara* spp. in American mink, accompanied by 95% CIs, in relation to: **a**, **b** study site; **c**, **d** year of American mink death; and **e**, **f** sex as predicted by the generalized linear models (GLM). F—female, M—male. Rug plots along the *x* axis show the data distribution.

Seroprevalence varied between the seven study sites (Fig. 3a, b; Tables 1, 2). In detail, the highest *Echinococcu*s spp. seroprevalence was found in SNP (28.9%) and in DNP (20.0%) and for both sites was significantly higher than in GR, BNP, and NNP, where seroprevalence was the lowest: 2.9%, 9.3%, and 11.1%, respectively (Tables 1, 2). Anti-*Toxocara* spp. antibodies were most prevalent in American mink from VR (72.2%), GR (65.7%), and SNP (42.3%). Seroprevalence was significantly higher in these sites than in WMNP, DNP, BNP, and NNP, where seroprevalence varied between 15.0 and 20.5% (Tables 1, 2). A comparison of seropositivity proportions in consequent years revealed significant (over three times) increase in *Toxocara* spp. seropositivity during 12 years of the study (Fig. 3d; Table 2). On the contrary, such dynamics were not observed among *Echinococcus* spp.-seropositive American mink (Fig. 3c; Table 2). Among 156 American mink seropositive for *Echinococcu*s spp., 94 were male (60.2%) and 62 were female (39.8%). A similar pattern was observed for *Toxocara* spp., where from among 239 seropositive animals 144 (60.3%) were male and 95 (39.7%) female; however, this relationship was not significant (Fig. 3e, f; Table 2).

**Table 2:** The analysis of explanatory variables in the generalized linear model (GLM) and their effects on the presence of *Echinococcus* spp. and *Toxocara* spp. Significant effects are marked by an asterisk.

Variables	Estimate	SE	*z* value	*P* value
*Toxocara*				
Intercept	− 282.64	87.25	− 3.24	0.00120**
Sex (M)	0.06	0.16	0.35	0.72382
Year	0.14	0.04	3.22	0.00128**
WMNP_DNP	− 0.01	0.35	− 0.04	0.97020
WMNP_GR	1.90	0.40	4.72	0.00000***
WMNP_SNP	1.20	0.32	3.75	0.00018***
WMNP_VR	2.66	0.55	4.87	0.00000***
WMNP_BNP	0.19	0.29	0.66	0.51182
WMNP_NNP	0.27	0.19	1.44	0.14936
GR_DNP	− 1.91	0.50	− 3.85	0.00012***
SNP_DNP	− 1.21	0.43	− 2.83	0.00465**
VR_DNP	− 2.68	0.61	− 4.37	0.00001***
BNP_DNP	− 0.21	0.40	− 0.51	0.61019
NNP_DNP	− 0.28	0.34	− 0.84	0.40177
SNP_GR	0.70	0.46	1.52	0.12784
GR_VR	0.77	0.65	1.17	0.24133
BNP_GR	1.71	0.46	3.68	0.00024***
NNP_GR	1.63	0.39	4.16	0.00003***
SNP_VR	1.47	0.60	2.45	0.01450*
BNP_SNP	1.00	0.39	2.60	0.00944**
NNP_SNP	0.93	0.31	3.01	0.00261**
BNP_VR	2.47	0.58	4.23	0.00002***
NNP_VR	2.39	0.54	4.43	0.00001***
BNP_NNP	0.08	0.28	0.27	0.78695
*Echinococcus*				
Intercept	− 31.83	93.65	− 0.34	0.73400
Sex (M)	− 0.04	0.18	− 0.21	0.83270
Year	0.02	0.05	0.32	0.74640
WMNP_DNP	0.18	0.31	0.58	0.55990
WMNP_GR	− 2.02	1.03	− 1.95	0.05130
WMNP_SNP	0.64	0.34	1.88	0.06010
WMNP_VR	− 0.51	0.76	− 0.67	0.50480
WMNP_BNP	− 0.71	0.36	− 1.96	0.05050
WMNP_NNP	− 0.52	0.21	− 2.53	0.01140*
DNP_GR	− 2.20	1.07	− 2.06	0.03910*
DNP_SNP	0.46	0.42	1.09	0.27670
DNP_VR	− 0.69	0.80	− 0.86	0.38800
DNP_BNP	− 0.89	0.43	− 2.05	0.04056*
DNP_NNP	− 0.70	0.32	− 2.21	0.02734*
GR_SNP	2.66	1.06	2.50	0.01249*
GR_VR	1.51	1.27	1.19	0.23620
GR_BNP	1.31	1.08	1.21	0.22640
GR_NNP	1.49	1.03	1.45	0.14850
SNP_VR	− 1.15	0.81	− 1.41	0.15734
SNP_BNP	− 1.35	0.46	− 2.93	0.00336**
SNP_NNP	− 1.16	0.34	− 3.38	0.00072***
VR_BNP	− 0.20	0.82	− 0.24	0.80900
VR_NNP	− 0.01	0.77	− 0.02	0.98600
BNP_NNP	0.18	0.37	0.50	0.61592

WMNP, Warta Mouth National Park; GR, Gwda River; DNP, Drawa National Park; SNP, Słowiński National Park; VR, Vistula River; BNP, Biebrza National Park; NNP, Narew National Park.

*P > 0.05; **P > 0.01; ***P > 0.001

**Figure 4:**
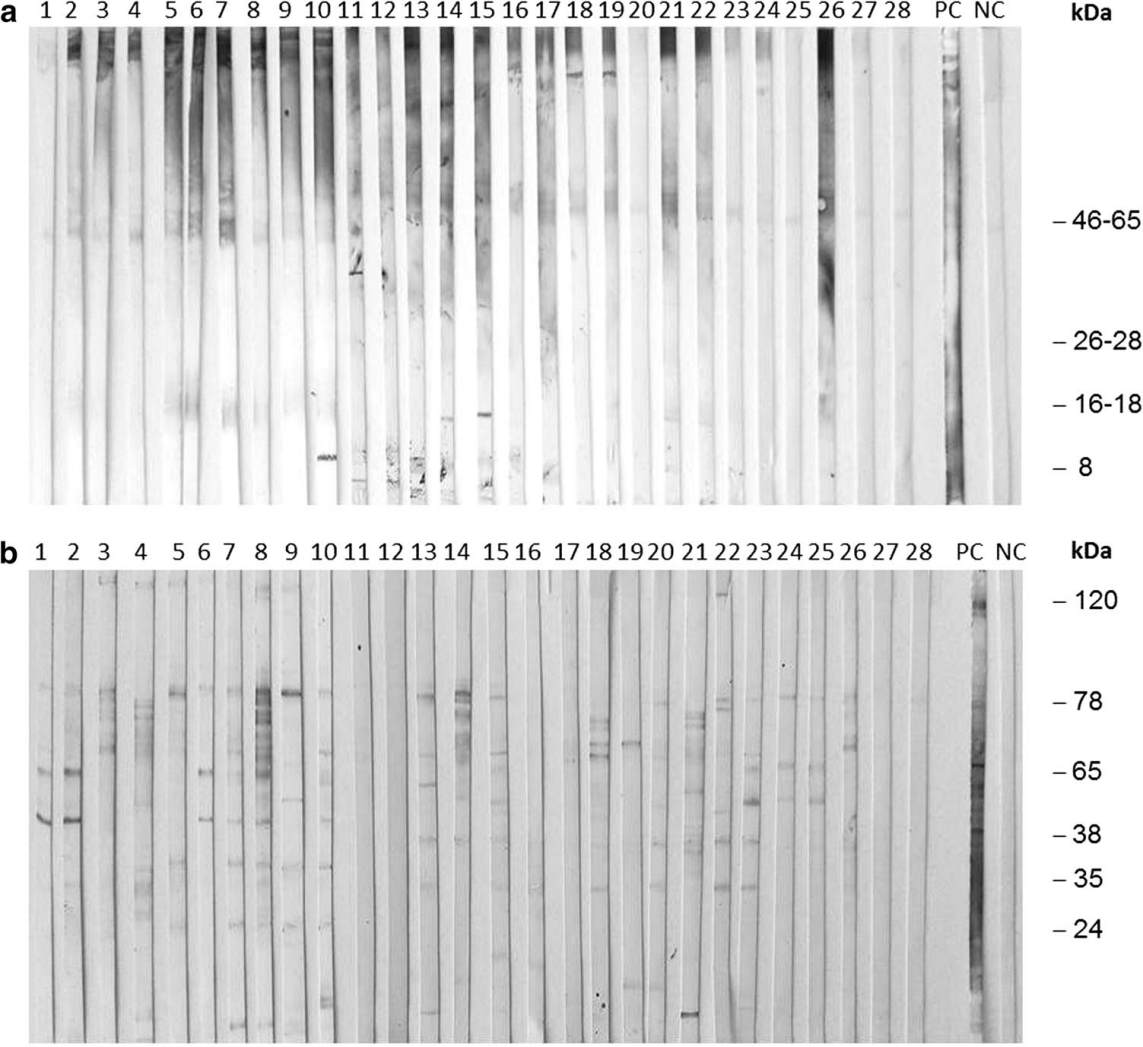
Western blot analysis of: **a***Echinococcus*-specific IgG antibodies in selected American mink samples. The lines represent: 1–28—selected American mink samples ELISA-positive for *Echinococcus*, PC—positive *E. multilocularis* control dog serum, NC—negative control dog serum. **b***Toxocara*-specific IgG antibodies in selected American mink samples. The lines represent: 1–28—selected American mink samples ELISA-positive for *Toxocara*, PC—positive *T. canis* control dog serum, NC—negative control dog serum.

The Western blot analysis proved the specificity of IgG antibodies detected in ELISA for both *Echinococcus* and *Toxocara* parasites. The examined samples showed specific bands for *Echinococcus* (8, 16–18, and 26–28 kDa) (Fig. 4a) and *Toxocara* (35, 38, and 65–78 kDa) (Fig. 4b). Additionally, the majority of samples showed bands of 46–65 kDa for *Echinococcus* and 24 and 120 kDa for *Toxocara*.

## Discussion

Our study revealed that American mink produce specific anti-*Echinococcus* spp. and anti-*Toxocara* spp. antibodies. The specific antibody presence confirms that this non-native invasive mammal species has been exposed to both studied zoonotic parasites in the wild. This—to the best of our knowledge—has never before been confirmed in the literature. Our parasitological investigation of American mink alimentary tracts revealed that neither *Echinococcus* spp. nor *Toxocara* spp. adults were found, which may support our hypothesis of American mink being paratenic rather than definitive hosts for either parasite. However, the above statement needs to be further supported by more detailed studies, because knowledge about the reservoir of AE in wildlife is patchy and focuses mainly on red fox (definitive host) infection (Bagrade et al. 2016; Karamon et al. 2014; Miller et al. 2016; Umhang et al. 2016). There is a lack of comprehensive data about the host status of American mink for *E. multilocularis*. The only one experimental study by Ooi et al. (1992) revealed that after double oral inoculation with 70,000 protoscoleces, no adult tapeworms were recovered from American mink intestines. The authors concluded that American mink cannot serve as a definitive host for *E. multilocularis* (Ooi et al. 1992). Parasitological studies of wild American mink have not revealed adult *E. multilocularis* tapeworms in neither Belarus (Shimalov and Shimalov 2001a) nor Poland (Kołodziej-Sobocińska et al. 2018; present study). In addition, adult forms of this tapeworm have not been found in other mustelid species, such as the stone marten (*Martes foina*), pine marten (*Martes martes*), European polecat (*Mustela putorius*), Eurasian badger (*Meles meles*), weasel (*Mustela nivalis*), or stoat (*Mustela erminea*) in Europe (Hurníková et al. 2009; Machnicka-Rowińska et al. 2002; Oksanen et al. 2016; Shimalov and Shimalov 2001b, 2002; Shimalov et al. 2000). Thus, we propose that the American mink serves as a paratenic host for *E. multilocularis*, since we revealed in the presented study the activation of its specific immune response, despite not finding adult tapeworms during necropsies. In wild definitive hosts, the occurrence of this tapeworm is mainly proved through the conduction of necropsies or the determination of eggs in faeces (Machnicka-Rowińska et al. 2002; Reiterová et al. 2006). Methods for detecting *E. multilocularis* coproantigen in definitive hosts have also been used (Deplazes and Eckert 1996; Machnicka et al. 2003); unfortunately, confirmation of echinococcosis remains difficult in intermediate/paratenic hosts. In wildlife, AE has only been reported in a few cases: muskrats (*Ondrata zibethicus*) and coypus (*Myocastor coypus*) (Miterpáková et al. 2006; Umhang et al. 2013). One study, which revealed *E. multilocularis* cysts in muskrats with 4.4% prevalence in Russia (Siberia, Selenga river), indicates the American mink as a source of muskrat infection, together with red foxes and domestic dogs (Masur and Fomina 2012).

The second studied parasite, *Toxocara* spp., can infect a wide range of companion, domestic, and wild animals as both definitive and paratenic hosts via multiple routes of transmission, producing long-lived, tissue-inhabiting larvae and resistant eggs that can survive in the external environment (Holland 2017). A recent experimental study by Klockiewicz et al. (2019a) revealed that American mink may be paratenic hosts for *T. canis* and *T. leonina*. In this study, tissue larvae were found in experimentally infected farm American mink; histopathological examinations of parenchymal organs and striated muscles revealed lesions resembling those observed in other paratenic hosts due to toxocarosis (Klockiewicz et al. 2019b). In addition, this infection was irrevocably confirmed by both ELISA and Western blot (Klockiewicz et al. 2019a). Several up-to-date parasitological studies of American mink have been conducted in Belarus (Shimalov and Shimalov 2001a), Spain (Torres et al. 2003), France (Torres et al. 2008), and Poland (Kołodziej-Sobocińska et al. 2018), but neither adult *Toxocara* spp. specimens nor eggs were detected, as in the present study. Among other mustelid species, pine marten in north-eastern and southern Poland has been found to be infected with *Toxocara cati* (Borecka et al. 2013; Górski et al. 2006). The difficulties with finding larvae specimens in the tissues of intermediate/paratenic hosts (such as humans) have led to the development of serological tests for use in toxocarosis diagnostics (Dziemian et al. 2008; Fillaux and Magnaval 2013). Used on rodent hosts, these methods deduced a 14.2% *T. canis* seroprevalence and showed ELISA to be more sensitive than PCR for detecting infection with the parasite (Krucken et al. 2017). Studies using immunodiagnostic methods have been carried out worldwide (Krucken et al. 2017; Li et al. 2017; Lassen et al. 2016; Klockiewicz et al. 2019a). Serological analysis also allows the easy detection of mixed infections in hosts. Our research showed that, on average, 6% of American mink are seropositive for both *Echinococcus* and *Toxocara* parasites.

As the American mink hosts many more parasite species (Hurníková et al. 2016; Kołodziej-Sobocińska et al. 2018; Torres et al. 2003, 2008), it likely plays a role in maintaining and transporting infectious agents during the colonization of new territories. The pronounced increase in *Toxocara* spp. seropositivity proportion over time is consistent with the pattern of parasite acquisition by non-native American mink in the course of the colonization of Poland (Kołodziej-Sobocińska et al. 2018). We did not observe such patterns for *Echinococcus* spp. seropositivity in American mink. This may be connected with an increase in mink density over time (Brzeziński et al. 2019), which results in American mink inhabiting areas closer to human development, this increasing the contact rate of American mink with dogs and cats (*Felis catus*). Dogs and cats are more involved in *Toxocara* spp. than in *Echinococcus* spp. transmission, which is mainly transmitted by wild carnivores, i.e. red fox or raccoon dog. In addition, we found great variation in the proportion of seropositive American mink in space; the highest seroprevalence of toxocarosis occurred in sites located in central Poland, while very low seroprevalence was seen in sites located in eastern and western Poland. This finding is difficult to explain; however, areas with a high prevalence of infected animals present a constant infection pressure for definitive and paratenic hosts. An opposite explanation may suggest that the abundance of *Toxocara* spp. in the environment had increased during the last years of the study. Further analyses of both spatial and temporal patterns may highlight the mechanisms of the circulation of both parasites in the environment.

Unravelling the role of non-native species in the epidemiology of pathogens is important to more sufficiently manage diseases. Such a role (in the maintenance and spread of pathogens) becomes even more crucial when the infectious agents can instigate serious diseases in humans (Duscher et al. 2017; Hurníková et al. 2016; Laurimaa et al. 2016). The American mink cannot transmit both parasites to humans by shedding parasite eggs, as it seems to be a paratenic host. However, American mink can be eaten by other predators who act as definitive hosts for *Echinococcus* spp. and/or *Toxocara* spp. including the wolf, Eurasian lynx, red fox, raccoon dog, and pets such as dogs and cats (Bryan et al. 2006; Errington 1937; Odden et al. 2006; Sepúlveda et al. 2014). As a result, high American mink densities in some areas (Bartoszewicz and Zalewski 2003) may sustain high infection risks in ecosystems.

In the case of *E. multilocularis*, it is thought that human AE is mainly present in areas with high densities of infected red foxes (Nahorski et al. 2013; Schweiger et al. 2007). We also hypothesized that the frequency of *Echinococcus* spp. seropositive in American mink should be higher in areas with a high infection prevalence of red fox. However, these data are not consistent in northern and north-western Poland, which have some of the lowest infection rates seen in red foxes (Karamon et al. 2014) (Fig. 1b), though relatively high seroprevalence in American mink as well as high numbers of confirmed human AE cases (Nahorski et al. 2013) (Fig. 1a, c). Similarly, non-native raccoon dogs have been found infected with *E. multilocularis* in north-western Poland, suggesting that wild species other that red fox may influence the occurrence of human cases (Machnicka-Rowińska et al. 2002; Karamon et al. 2016). Even if a species serves as a paratenic host, as American mink does, it may also serve as a prey item for definitive hosts like red fox or raccoon dog. In this way, paratenic hosts may enhance the probability of disease transmission (Wobeser 2006). This causes the circulation of the parasite in the wild, and as a result, the parasite may be maintained at high abundances in the environment and also affect infection levels in humans. More studies are needed to investigate the source of human infection in these areas, including the role of American mink and other non-native species.

## Conclusions

To conclude, our study shows that the non-native invasive American mink is a species exposed to two serious zoonotic parasite genera—*Echinococcus* and *Toxocara*. Relatively high seropositivity of American mink in some feral populations indicates that the species may have a role in the maintenance of these parasites in wildlife. As the American mink may serve as a paratenic host, it can be an important link in the transmission routes of these parasites. Spatial distribution and high American mink densities occurring on colonized areas result in higher seropositivity of American mink over time and space; as a result, this may cause the host to maintain higher levels of infection risk for both wild and domestic definitive hosts, thus increasing the risk of human infection through contact with the environment contaminated with *Echinococcus* spp. and/or *Toxocara* spp. eggs.
